# The Composition of Microbial Communities in Six Streams, and Its Association With Environmental Conditions, and Foodborne Pathogen Isolation

**DOI:** 10.3389/fmicb.2020.01757

**Published:** 2020-07-29

**Authors:** Taejung Chung, Daniel L. Weller, Jasna Kovac

**Affiliations:** ^1^Department of Food Science, The Pennsylvania State University, University Park, PA, United States; ^2^Microbiome Center, Huck Institutes of the Life Sciences, The Pennsylvania State University, University Park, PA, United States; ^3^Department of Food Science, Cornell University, Ithaca, NY, United States

**Keywords:** surface water, agricultural water, microbial communities, food safety, produce safety

## Abstract

Surface water used for produce production is a potential source of pre-harvest contamination with foodborne pathogens. Decisions on how to mitigate food safety risks associated with pre-harvest water use currently rely on generic *Escherichia coli*-based water quality tests, although multiple studies have suggested that *E. coli* levels are not a suitable indicator of the food safety risks under all relevant environmental conditions. Hence, improved understanding of spatiotemporal variability in surface water microbiota composition is needed to facilitate identification of alternative or supplementary indicators that co-occur with pathogens. To this end, we aimed to characterize the composition of bacterial and fungal communities in the sediment and water fractions of 68 agricultural water samples collected from six New York streams. We investigated potential associations between the composition of microbial communities, environmental factors and *Salmonella* and/or *Listeria monocytogenes* isolation. We found significantly different composition of fungal and bacterial communities among sampled streams and among water fractions of collected samples. This indicates that geography and the amount of sediment in a collected water sample may affect its microbial composition, which was further supported by identified associations between the flow rate, turbidity, pH and conductivity, and microbial community composition. Lastly, we identified specific microbial families that were weakly associated with the presence of *Salmonella* or *Listeria monocytogenes*, however, further studies on samples from additional streams are needed to assess whether identified families may be used as indicators of pathogen presence.

## Introduction

In 2015, United States agriculture used over 118 million gallons of water per day for irrigation, with more than half of all withdrawals originating from surface water sources (Dieter et al., 2018). These waters represent an important source of agricultural water used in fresh produce production, making microbial quality of surface water used for produce production a critical food safety concern (Doyle and Erickson, 2008; Ceuppens et al., 2014; Jones et al., 2014; Weller et al., 2015a; Markland et al., 2017). Recent multistate foodborne outbreaks associated with fresh produce have emphasized the need for improved detection and mitigation of the food safety risks associated with the surface water use (CDC, 2012; Markland et al., 2017). One of the examples is a high-profile *E. coli* O157:H7 outbreak in 2018 associated with romaine lettuce that resulted in 62 cases of foodborne illness, including 25 hospitalizations (FDA, 2019b). During this outbreak investigation, the outbreak strain was detected in a sediment sample from a reservoir that provided water to the implicated farms, suggesting that the agricultural water may have been a potential source of contamination (Gelting et al., 2011; Mody et al., 2011; FDA, 2019b). This and other multistate foodborne disease outbreaks associated with fresh produce have increased concerns related to the use of surface water for produce production and processing (Gelting et al., 2011).

Current United States industry standards [e.g., Leafy Greens Marketing Agreement (LGMA, 2019)] and the proposed federal regulations (FDA, 2017) use generic *E. coli*-based tests to manage food safety risks related to pre-harvest water use. Several studies reported a variable or weak correlation between generic *E. coli* levels and the detection or concentration of foodborne pathogens (Haley et al., 2009; Payment and Locas, 2010; McEgan et al., 2013; Draper et al., 2016; Pachepsky et al., 2016; Falardeau et al., 2017; Truchado et al., 2018) or their virulence genes (Topalcengiz et al., 2017) in agricultural water, whereas several studies indicated strong correlation (Goyal et al., 1977; Mansilha et al., 2010; Xiao et al., 2013; Poma et al., 2016). Furthermore, *E. coli* is typically used as an indicator of fecal contamination and not as an index for specific foodborne pathogens. Since many foodborne pathogens can survive and grow in non-host environments, pathogen contamination of agricultural water may not always be associated with fecal contamination. One such example is *L. monocytogenes*, a foodborne pathogen of concern and a free-living soil microorganism that is broadly distributed in the natural environment, and is not always associated with fecal contamination (Weller et al., 2015a, 2019). Even in cases when the pathogen contamination does originate from a fecal source, some foodborne pathogens and generic *E. coli* have been found to respond to environmental conditions differently. For instance, *Salmonella* was shown to survive better compared to *E. coli* in non-host environments (Winfield and Groisman, 2003; Pachepsky et al., 2016). These limitations of using generic *E. coli*-based water quality tests, as well as spatial and temporal variation in microbial water quality (McEgan et al., 2013; Chidamba et al., 2016; Draper et al., 2016; Nguyen et al., 2016; Falardeau et al., 2017; Pachepsky et al., 2018) are illustrative of the need for improved methods of identifying and mitigating the food safety risks associated with the pre-harvest water use. Metagenomics-based microbial ecology studies may provide one such alternative by which specific taxa (or set of taxa) whose presence or absence is associated with increased risk for contamination of surface water with foodborne pathogens may be identified to provide potential targets for new tests. Development of alternative tests therefore necessitates a better understanding of the surface water microbial ecology, including changes in microbial community structure over time and space, and with respect to environmental factors and foodborne pathogens.

Studies reported to date found spatial and temporal variation in the composition of microbial communities collected from surface water (Van Rossum et al., 2015; Wang et al., 2016, 2018, 2019; Payne et al., 2017). While core microbial communities were identified across samples collected from the same stream waterways, the relative abundance of core taxa varied over time and space (Van Rossum et al., 2015; Payne et al., 2017). Furthermore, seasonal and environmental factors, such as weather, land use adjacent to sampling sites, and concentration of certain chemicals have been shown to affect the microbiome composition (Van Rossum et al., 2015; Payne et al., 2017; Wang et al., 2018). However, limited data characterizing associations between microbial community composition and foodborne pathogen isolation from agricultural water samples are available (Allard et al., 2019). Thus, the goal of this small-scale study was to (i) characterize bacterial and fungal communities in surface waters collected from six streams in the Northeastern United States over time, and to (ii) investigate potential associations between microbial community composition, environmental factors (e.g., turbidity, water temperature, rainfall), and foodborne pathogen isolation.

## Materials and Methods

### Sample Collection

Moore swabs (*n* = 68) were collected between June and August 2017 from 6 streams (A, B, C, D, E, and F) in New York state as described in Weller et al. (2020). Briefly, each stream was sampled for three non-consecutive weeks. At the beginning of each week, a Moore swab was placed in the stream and let float in the water. After 24 h, the swab was collected, and a second swab was placed in the stream. This was repeated for up to seven consecutive days, resulting in a total of 11–13 collected samples per stream. After sample collection, each swab was placed in a sterile Whirl-Pak bag and stored at 4°C until processing, which occurred within 6 h after sample collection.

### Sample Processing

After hand-massaging each swab for 1 min, the swab was pressed and 100 ml of the stream water were extracted using a sterile serological pipette. The 100 ml were then transferred to two 50-ml sterile conical tubes. Due to high levels of sediment that inhibited sample filtration, each tube was centrifuged at 4,000 × *g* at 4°C for 5 min. The water fraction was then filtered through a 0.45 μm 250 ml analytical filter system (VWR, Radnor, PA, United States), and transferred to DNeasy PowerWater Kit (Qiagen, Hilden, Germany) filter collection tube. The biomass retained on the filter is here referred to as the *water fraction.* The sediment pellet that formed after centrifugation was kept and is here referred to as the *sediment fraction*. The sediment and water fractions were stored at −80°C until DNA extraction. They were treated as separate samples in all downstream sequencing and data analyses.

### Metadata Acquisition, Waterway Enrollment, and Foodborne Pathogen Presence Data

Remotely sensed stream and land use data were obtained from publicly-available datasets (EWEM, 2017; MRLC, 2017; USDA, 2017; USGS, 2017) to facilitate waterway enrollment (Weller et al., 2020). These data were used to identify watersheds that were >15 km^2^ in area, and <400 m from a field where produce was grown in ≥4 of the last 8 years (2009–2016). Publicly accessible sites along these watersheds were identified, and six sites were randomly selected for enrollment so that all enrolled sites were in non-overlapping watersheds (Weller et al., 2020).

### Metadata Collection

All physicochemical environmental water quality data as well as all pathogen data used in the analyses here had been reported by Weller et al. (2020). Briefly, physicochemical water quality, air temperature, and flow data were collected each time a Moore swab was deployed or collected (see Supplementary Table S1 for a data summary). Dissolved oxygen, conductivity, pH, and water temperature were measured at the time of the sample collection in the field using a Hach HQ40D Multi Meter (Hach, Loveland, CO, United States). Flow rate was also measured in field using a flow meter (Global Water Instrumentation Inc., Cordova, CA, United States). Turbidity was measured in the lab using a turbidimeter (Hach).

### DNA Extraction

Extraction of DNA from water and sediment fractions of each sample was performed using a DNeasy PowerWater DNA isolation kit or DNeasy PowerSoil DNA isolation kit (Qiagen), respectively, per manufacturers’ instructions. The extracted DNA was then eluted with 100 μl of 10 mM Tris elution buffer provided by the manufacturer. Concentration and purity of DNA in each sample was assessed using a NanoDrop One UV-Vis spectrophotometer (Thermo Fisher Scientific, Madison, WI, United States). All sample of extracted DNA were stored at −80°C until PCR amplification.

### PCR Amplification and Illumina Sequencing of the 16S rDNA V4 and ITS2

The extracted DNA was adjusted to a concentration of 10 μg/ml double-stranded DNA using a Qubit dsDNA High Sensitivity Assay Kit (Life Technologies). Samples with a standardized concentration were used as templates in PCR amplification of bacterial 16S rDNA V4 and fungal Internal Transcribed Spacer 2 (ITS2) sequences. Primers 515FB (GTGYCAGCMGCCGCGGTAA) and 806RB (GGACTACNVGGGTWTCTAAT) were used for amplification of the V4 sequence of the 16S rRNA gene (Walters et al., 2016). Primers ITS9F (GAACGCAGCRAAIIGYGARV) and ITS4R (TCCTCCGCTTATTGATATGC) were used for amplification of the ITS2 region (Ihrmark et al., 2012). PCR for each amplification was performed in 20 μl containing 10 μl of KAPA HiFi HotStart ReadyMix PCR Kit (Kapa Biosystems), 1 μl of each, the forward and reverse primers in concentration of 10 μM, 1 μl of the template DNA in concentration of 10 μg/μl and 7 μl of a PCR-grade ultrapure water. Thermal cycling conditions were optimized based on the Illumina 16S rRNA gene metagenomic sequencing library preparation protocol (Illumina, 2013), and Joint Genome Institute iTag Sample Amplification QC SOP (Daum, 2016). For the 16S rRNA V4 sequence, an initial denaturation of 2 min at 98°C, followed by 25 cycles of 20 s at 98°C, 20 s at 56.5°C, and 25 s at 72°C, and a final step at 72°C for 5 min was used. For ITS2 amplification, an initial denaturation of 5 min at 98°C, followed by 30 cycles of 45 s at 95°C, 60 s at 50°C, and 60 s at 72°C, and a final step at 72°C for 5 min was performed. PCR amplicons were visualized using gel electrophoresis. A total of 114 DNA samples extracted from 68 samples of sediment fractions and 46 samples of water fractions resulted in successful PCR amplification, as confirmed by gel electrophoresis. Insufficient amount of DNA was obtained from 22 water fraction samples, resulting in no PCR amplification. The expected PCR amplicon size was confirmed using 2100 Bioanalyzer (Agilent) on sets of 12 pooled samples. After this quality control step that was carried out by the Penn State Huck Institutes of the Life Sciences’ Genomics Core Facility, PCR amplicons were submitted for library preparation and Illumina sequencing to the core facility. Amplicons were barcoded and cleaned-up following the Illumina 16S rRNA amplicon library preparation guide (Illumina, 2013). Libraries were quantified using quantitative PCR and normalized accordingly. Normalized libraries were pooled, denatured, spiked with PhiX and sequenced on an Illumina MiSeq platform at the Penn State Huck Institutes of the Life Sciences Genomics Core Facility. A 500 cycle V2 kit (Illumina) was used to obtain 250 bp paired-end reads. The targeted number of reads per sample was 40,000 bp per library. Sequences were deposited in the NCBI SRA database under the BioProject accession number PRJNA528961 (Supplementary Table S2).

### Sequence Read Quality Control, Assembly and Taxonomic Classification

Raw sequence reads were demultiplexed and further processed using Mothur v.1.40 with default settings and the parameters provided by the Mothur MiSeq SOP unless noted otherwise (Schloss et al., 2009; Kozich et al., 2013). The sequence analysis workflow is described in the R file deposited on GitHub: https://github.com/tuc289/SurfaceWaterMicrobiome/blob/master/Script.R. Briefly, low-quality bases were removed, and read pairs assembled into contigs. Contigs that were longer or shorter than the expected length of 282 bp for 16S rRNA V4 or were out of the 265 to 420 bp range for ITS2 were removed. Contigs were aligned to the SILVA 16S rRNA reference database (SILVA 132 release, July 2017) for bacteria (Glöckner et al., 2017) and ITS UNITE database (UNITE Mothur release, v 7.2, December 2017) for fungi (Koljalg et al., 2013). Contigs that remained unmapped, as well as chimeric contigs were discarded (Rognes et al., 2016). Operational taxonomic units (OTUs) were then generated using a 97% sequence similarity threshold (Schloss and Handelsman, 2005).

### Estimated Sample Richness and Alpha Diversity Analysis

To visualize the relationship between sequencing depth and observed species richness (i.e., the cumulative number of unique OTUs observed in each sample), rarefaction curves were constructed by random subsampling of reads from each library, and plotted using ggplot2 package version 3.1.0) in R version 3.5.2 (Wickham, 2009). Total richness, which accounts for both the observed and unobserved OTUs likely to be present in a samples, in each sample was then estimated using Chao1 richness estimator from the spadeR package version 0.1.1 (Chao et al., 2015) and compared with the observed richness. An alpha diversity index was calculated for each sample’s water and sediment fraction to investigate the within-sample diversity of bacterial and fungal communities. Alpha diversity was assessed by calculating the Inverse Simpson index (Simpson, 1949), that reflects sample richness and evenness. The Inverse Simpson index was calculated based on the OTUs normalized using relative log expression (RLE) method (McMurdie and Holmes, 2014; Maza, 2016). The distribution of the Inverse Simpson index in samples from each stream were obtained and plotted using the phyloseq package v 1.16.1 (“plot_richness”) and ggplot2 version 3.1.0 in R (Wickham, 2009; McMurdie and Holmes, 2013). Differences in alpha diversity observed in samples from different streams were assessed statistically using a Kruskal-Wallis test and a Dunn *post hoc* test (McKight and Najab, 2010; Dinno, 2015).

### Beta Diversity Analysis

Beta-diversity was calculated based on RLE-normalized OTU table to investigate the among-sample diversity of bacterial and fungal microbial communities detected in sediment and water fractions, respectively (Whittaker, 1960). Beta diversity was assessed by calculating weighted UniFrac distances that account for the distance between each pair of samples, with respect to the relative abundance of each taxonomic family and their phylogenetic relationship (Lozupone et al., 2010). Principle coordinate analysis (PCoA) was carried out using weighted UniFrac distances to visualize the ordination and clustering among the samples. The first two principal coordinates were plotted using phyloseq and ggplot2 packages in R (Wickham, 2009; McMurdie and Holmes, 2013). Samples were color-coded to visually assess putative clustering of samples based on the (i) sample fraction (sediment or water fraction), (ii) stream (A through F), and (iii) foodborne pathogen presence (isolated *Salmonella* or *L. monocytogenes*).

### Identification of Factors Associated With the Composition of Bacterial and Fungal Communities

Permutational Multivariate Analysis of Variance (PERMANOVA) using distance matrices was carried out to identify potential associations between microbial community composition and factors of interest identified through PCoA clustering (by sample fraction and stream). The analysis was carried out using the UniFrac distance matrix for each pair of samples and the “adonis” function in the R vegan package (Oksanen et al., 2018). Pairwise PERMANOVA was carried out to determine if microbial community composition varied significantly between samples collected from six different streams. The analysis was carried out with 999 permutations using the “pairwise.adonis” function implemented in the R package pairwiseAdonis version 0.0.1 (Oksanen et al., 2018; Martinez Arbizu, 2019). For pairwise PERMANOVA, *p* values were corrected for multiple comparisons using Bonferroni correction. The total number of comparisons was 15 and the corrected alpha value was 0.003 (Bonferroni, 1936).

Differential abundance test implemented in QIIME2 plug-in Songbird and Qurro were used to identify families that were differentially abundant between sediment and water fractions (Bolyen et al., 2019; Morton et al., 2019). Wald test was used to test the significance of differential abundance of genera *Yersinia* and *Thorsellia* from the family *Enterobacteriaceae* (Wald, 1945).

Canonical correspondence analysis (CCA) was used to assess the relationship between microbial community composition, and environmental factors. CCA was performed using the “cca” function in the vegan package (Oksanen et al., 2018). Seven environmental measurements were used in the analysis: pH, dissolved oxygen, flow rate, air temperature, water temperature, conductivity, and turbidity. Permutation analysis was used to identify specific environmental factors that were potentially associated with the composition of bacterial and fungal microbial communities. Repeated measures study design (i.e., collection of multiple samples from the same sampling site) was accounted for in the CCA analysis by incorporating the sampling site (i.e., stream) as a within-group factor when testing for associations between environmental factors and microbial community composition. Analyses were carried out separately for bacterial and fungal communities.

Correlation between the abundance of each family that was present in relative abundance of 2.5% or higher and each environmental factor were then calculated using repeated measures correlation method implemented in the R package rmcorr version 0.3. This allowed us to estimate a common correlation slope that accounted for repeated sampling from individual streams (Bakdash and Marusich, 2017). Independently of rmcorr analyses, we also examined potential Spearman’s correlation betweens individual environmental factors using the R package corrplot version 0.85 (Weller et al., 2015b, 2020).

### Evaluating Potential Associations Between Microbial Community Composition and Foodborne Pathogen Isolation

Random forest analysis was performed on RLE-normalized OTU table to identify and rank variables (i.e., microbial families) whose presence and relative abundance were associated with foodborne pathogen presence (Breiman, 2001; Strobl et al., 2008; Statnikov et al., 2013). The pathogen data used here are reported in Weller et al. (2020), and include data on the presence or absence of viable *Salmonella* and *Listeria monocytogenes* in each sample (Data summary available in Supplementary Table S3). *Salmonella* and *L. monocytogenes* positive samples were identified using culture-based enrichment methods (Weller et al., 2020). Presumptive *Salmonella* and *L. monocytogenes* positive colonies were confirmed as *Salmonella* or *L. monocytogenes* by PCR amplification of the *invA* and *sigB* genes, respectively (Nightingale et al., 2005; Kim et al., 2007; Den Bakker et al., 2010; Bundrant et al., 2011; Weller et al., 2020). Two random forest analyses were performed per pathogen, using pathogen detection result (present or absent) as an outcome and either bacterial or fungal OTU table as an input. Conditional variable importance was calculated and used to (i) identify variables that were not associated with pathogen detection (families with a variable importance ≤0), and (ii) rank variables that were associated with pathogen detection (families with a variable importance >0) (Strobl et al., 2008). All identified variables were then normalized by min-max normalization (Jain and Bhandare, 2011). An additional analysis with a sampling site added as a variable was ran to evaluate the effect of repeated sampling from the same six streams over time.

## Results

A total of 68 Moore swabs were selected and fractionated into 68 sediment fractions and 68 water fractions. DNA was successfully extracted and PCR-amplified for 68 samples of sediment fractions and 46 samples of water fractions, which were then sequenced. Insufficient amount of DNA was obtained from 22 water fraction samples, resulting in no PCR amplification. A median of 30,844 reads per sample were obtained for 16S rRNA V4 PCR amplicons (*n* = 114) [min = 5,272, max = 47,078, standard deviation (SD) = 8,371.36) and a median of 33,297 reads for ITS2 PCR amplicons (*n* = 114) (min = 5,700, max = 79,301, SD = 13,814]. Reads that passed the quality control were assigned operational taxonomic units (OTUs). A median of 19,437 OTUs per sample was obtained for 16S rRNA V4 PCR amplicons (min = 2,375, max = 21,889, SD = 5,418) and a median of 18,737 for ITS2 PCR amplicons (min = 4,207, max = 57,051, SD = 10,643).

**FIGURE 1:**
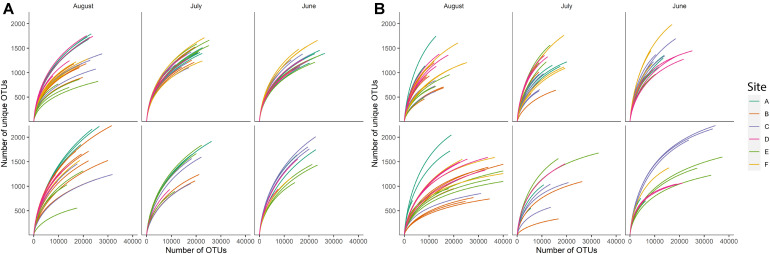
Rarefaction curves for **(A)** bacterial and **(B)** fungal species richness observed in sample sediment fractions (top row) and sample water fractions (bottom row) based on the month of sampling. Each color indicates a different sampling site (*n* = 6).

Rarefaction curves for individual samples suggested uneven richness among samples (Figure 1). Using a richness estimator, we determined that a median of 72.2% (min = 47.69%, max = 77.9%) and 77.9% (min = 68.62%, max = 86.27%) of the total estimated bacterial and fungal richness, respectively, was observed in the sequenced samples at the sequencing depth used in the present study (Chao et al., 2015). Sample F25 from sediment bacterial community was excluded from further analyses since observed species richness was 47.69% of estimated richness, suggesting that this sample was severely under-sequenced. The observed species richness in all remaining sequences (135 sediment fraction sequences and 92 water fraction sequences) accounted for more than 60% (arbitrarily determined threshold) of estimated richness.

### Microbial Communities Differed Among Sediment and Water Fractions, as Well as Among Streams

**FIGURE 2:**
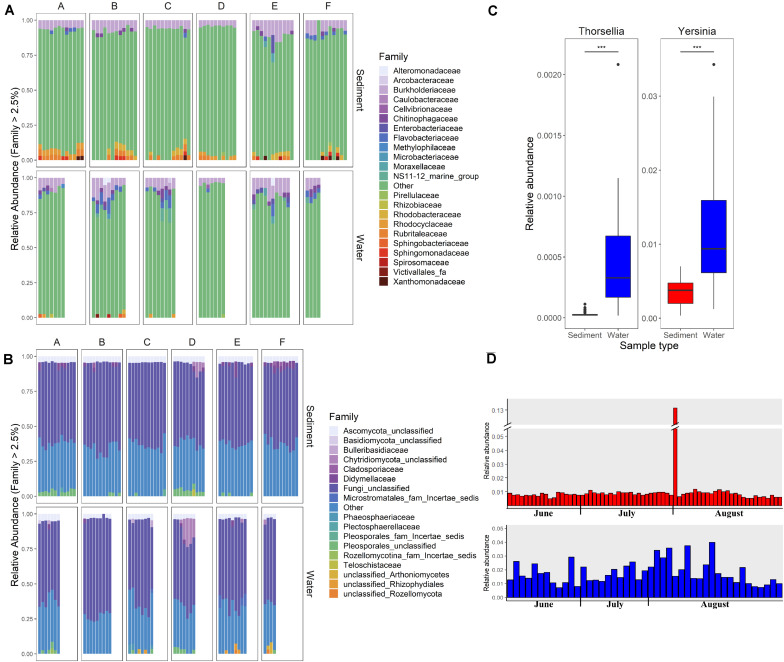
Relative abundance of **(A)** bacterial and **(B)** fungal families in sediment and water fractions of collected water samples. Each column represents one sample. Letters A, B, C, D, E, and F indicate six different sampled streams. Families with relative abundance of less than 2.5% are shown under the category “Other.” **(C)** Relative abundance of the predominant genera within Enterobacteriace family in sediment and water fractions of collected samples (Wald test, ***p < 0.001). **(D)** Relative abundance of *Enterobacteriace* in individual sediment and water fraction samples.

Differences in the overall microbial community composition between sediment and water fractions were investigated through the comparison of relative abundance of different taxa in each sample (Figures 2A,B), and beta diversity analysis using principal coordinate analysis (PCoA). Two bacterial families (*Chitinophagaceae* and *Burkholderiaceae*) and two fungal families (unclassified families from Pleosporales and from Ascomycota) represented the core microbial communities across all streams (Figures 2A,B). Generally, *Burkholderiaceae*, *Flavobacteriaceae*, *Chitinophagaceae*, *Rubritaleaceae*, and *Rhodobacteriaceae* were the most common and most abundant bacterial families in sediment and water fractions, while unclassified families from Pleosporales and from Ascomycota, *Bulleribasidiaceae*, and *Didymellaceae* were most common and most abundant fungal families across sediment and water fractions.

**FIGURE 3:**
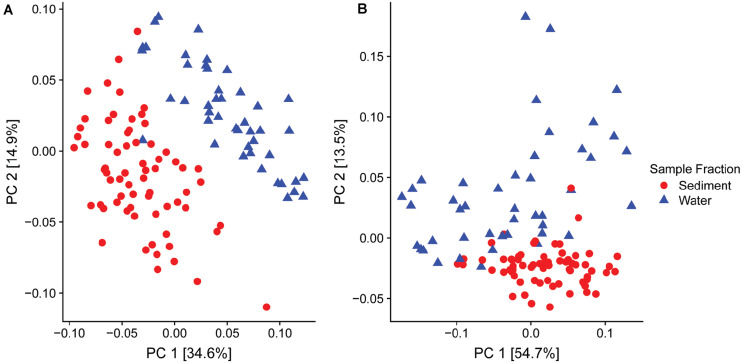
Principal Coordinate Analysis (PCoA) based on the UniFrac distances for **(A)** bacterial and **(B)** fungal families found in sediment (*n* = 68, red dots) and water (*n* = 46, blue dots) fractions of collected water samples.

Of particular interest was the variable relative abundance of *Enterobacteriaceae* in water fractions of collected samples over time (Figures 2C,D). The family of *Enterobacteriaceae* identified in the present dataset was predominantly comprised of *Yersinia* spp. and *Thorsellia* spp. These genera were detected in a significantly higher relative abundance in water fractions compared to sediment fractions (*p* = 1.005^–4^ and *p* = 1.529^–37^, respectively), according to the Wald test (Figure 2C). Furthermore, separate PCoA plots generated for bacterial (Figure 3A) and fungal (Figure 3B) communities indicated that the composition of bacterial and fungal communities was generally different in sediment fractions compared to the water fractions. Significant differences in microbial family composition were also confirmed using PERMANOVA (Supplementary Table S4).

**FIGURE 4:**
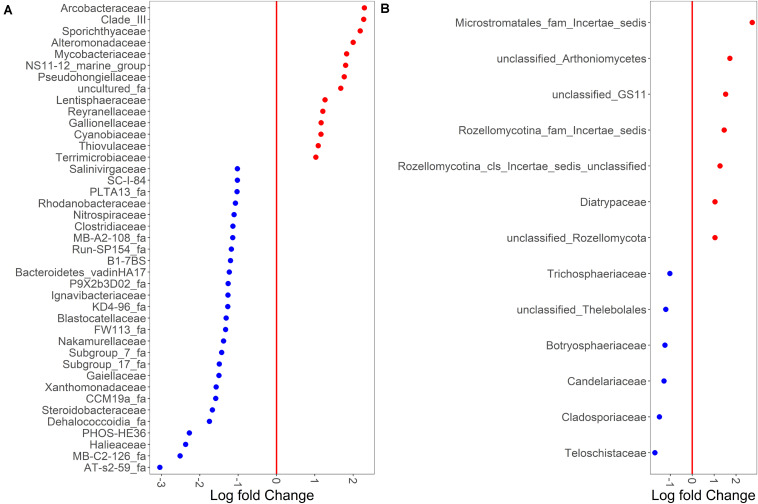
Binomial differential abundance of microbial families between **(A)** bacterial and **(B)** fungal communities of surface water samples.

Despite sample type-clustering, no family was uniquely present in either the sediment or water fractions. However, using a binomial test for differential abundance we identified bacterial families with different relative abundance in sediment fractions compared to water fractions. The Figure 4A and Supplementary Table S5 show families with significantly and at least 10-fold different relative abundance of families between water and sediment fractions. We identified 13 bacterial families with at least 10-fold higher relative abundance in sediment fractions and 27 bacterial families with at least 10-fold higher relative abundance in water fractions (Figure 4B and Supplementary Table S5). Specifically, *Sporichthyaceae*, *Alteromonadaceae*, *Mycobacteriaceae*, *Pseudohongiellaceae*, *Arcobacteraceae* and five unidentified families were the top ten families that were present in higher relative abundance in the sediment fractions. The *Gaiellaceae*, *Xanthomonadaceae*, *Steroidobacteraceae*, *Halieaceae*, and six unidentified families were the ten families with higher relative abundance in water fractions. In terms of fungal families, 13 families were differentially abundant (at least 10-fold) between sediment and water fractions (Figure 4B and Supplementary Table S6). Seven fungal families, which were mostly unclassified families under Microstromatales, Arthoniomycetes, Rozellomycotina except *Diatrypaceae*, had higher relative abundance in the sediment fractions while six families (*Teloschistaceae, Cladosporiaceae, Candelariaceae, Botryosphaeriaceae, Trichosphaeriaceae*, and unclassified family from Thelebolales) had higher relative abundance in water fractions.

**FIGURE 5:**
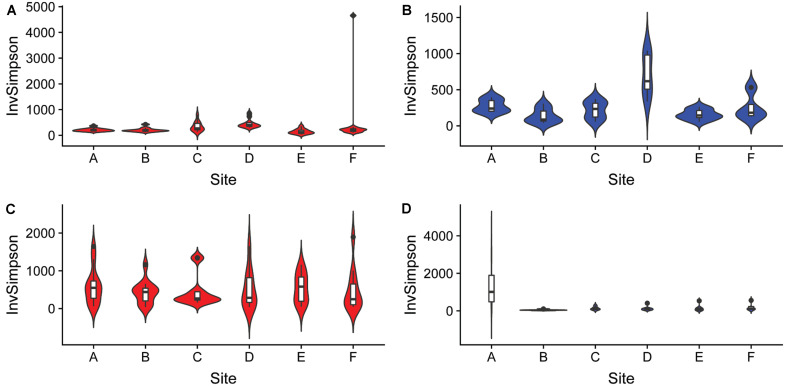
Alpha diversity of **(A,B)** bacterial and **(C,D)** fungal communities found in samples collected from six different streams using the Inverse Simpson index. The analysis included sediment (*n* = 68, red) and water (*n* = 46, blue) fractions of collected water samples.

The differences in microbial communities were observed not only among sediment and water fractions, but also among sampling sites that were located at different streams (one site per stream). For example, the alpha diversity of bacterial communities identified in both sediment and water fractions differed among samples collected from different streams (Figure 5 and Supplementary Table S7), as determined using the Kruskal-Wallis test. Specifically, the alpha diversity of bacterial communities found in sediment fractions from stream A and D; B and D; C and E; and D and F were significantly different. Streams B and D had significantly different alpha diversity of bacterial communities in water fractions. In terms of fungal communities, stream A and B; and A and E had significantly different alpha diversity in water fractions. Samples collected from different streams also had different bacterial (*p* < 0.001) and fungal (*p* = 0.001) community compositions according to Dunn’s test. The outcome of the pairwise PERMANOVA analysis suggested that the composition of bacterial communities in the sediment fractions was significantly different among all six sampled streams (Table 1). Samples from thirteen of 15 tested pairs of streams were significantly different in terms of fungal community composition in sediment fractions. Ten out of 15 tested pairs, and 11 out of 15 tested pairs were significantly different in terms of the composition of bacterial and fungal communities in the water fractions, respectively (Table 1). To hypothesize potential drivers of these differences between sites, we descriptively compared the land use upstream of the sampling sites (Supplementary Table S8). Statistical analyses were not performed to examine these relationships due to the small number of stream samples and insufficient power to detect spatial signals in the data. These observations should therefore be considered as speculative and can only serve as means of hypothesis generation.

**TABLE 1:** Differences in bacterial and fungal communities’ composition among different streams.

**Sampling site**	**Bacterial communities^a^**		**Fungal communities^a^**	
	
	**Sediment**	**Water**	**Sediment**	**Water**
A vs. B	**> 0.015**	**> 0.015**	**> 0.015**	**> 0.015**
A vs. C	**> 0.015**	**0.03**	**> 0.015**	**0.03**
A vs. E	**> 0.015**	**> 0.015**	0.06	**0.03**
A vs. F	**> 0.015**	0.06	**> 0.015**	**0.045**
A vs. D	**> 0.015**	**0.045**	**> 0.015**	**> 0.015**
B vs. C	**> 0.015**	**0.045**	0.075	**> 0.015**
B vs. E	**> 0.015**	**> 0.015**	**> 0.015**	**0.03**
B vs. F	**> 0.015**	0.105	**> 0.015**	0.075
B vs. D	**> 0.015**	**0.03**	**> 0.015**	**> 0.015**
C vs. E	**> 0.015**	0.885	**> 0.015**	0.285
C vs. F	**> 0.015**	0.465	**> 0.015**	1
C vs. D	**> 0.015**	**> 0.015**	**> 0.015**	**> 0.015**
E vs. F	**0.03**	0.66	**0.03**	1
E vs. D	**> 0.015**	**> 0.015**	**> 0.015**	**> 0.015**
F vs. D	**> 0.015**	**> 0.015**	**> 0.015**	**> 0.015**

*^*a*^Bonferroni-corrected *p*-values obtained by pairwise PERMANOVA analyses of weighted UniFrac distances between pairs of sampling sites. Pairs of sites with significantly different microbial communities (*p* < 0.05) are indicated in bold type.*

The predominant land cover in all six watersheds was forest-wetland cover (i.e., National Land Cover Classes (NLCD) 41–43, 52, 90 and 95; Supplementary Table S8). However, 0–250 m upstream of the sampling sites A and D the predominant land cover was developed (e.g., high impervious cover; NLCD classes 21–24; Supplementary Table S8), while the predominant land cover immediately upstream of sampling site B was forest-wetland cover. The predominant land cover immediately upstream of sites C, E, and F was agricultural (e.g., pasture, hay; NLCD classes 81–82; Supplementary Table S8). Based on observations noted during sample collection, dairy, produce, and hobby farms were visible immediately upstream of and adjacent to sampling sites C and F. The sampling site E was adjacent to a small industrial complex and was part of a public-right-of-way; dogs were observed roaming freely throughout this area. Streams A and D were near urban areas, while site B was immediately downstream of several large protected areas (e.g., state parks, state forests). The predominant bottom substrates in streams A, C, E, and F were rocky (e.g., gravel and cobble), stream B was sand, and stream D was organic matter. High amounts of silt and organic matter was also present along the bottom of stream B, while large boulders and clay were present along the bottom of stream D immediately upstream of the sampling site due to the presence of a road crossing.

### Several Environmental Factors Were Associated With the Composition of Microbial Communities

**FIGURE 6:**
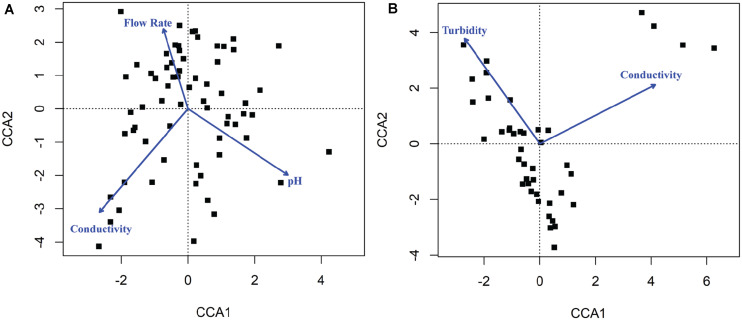
Canonical correspondence analysis (CCA) plot indicating physicochemical properties that were correlated with the composition of **(A)** bacterial communities found in sediments and **(B)** fungal communities found in water. The blue arrows and labels correspond to the physicochemical factors that were significantly associated with the composition of microbial communities in samples shown as black dots.

**FIGURE 7:**
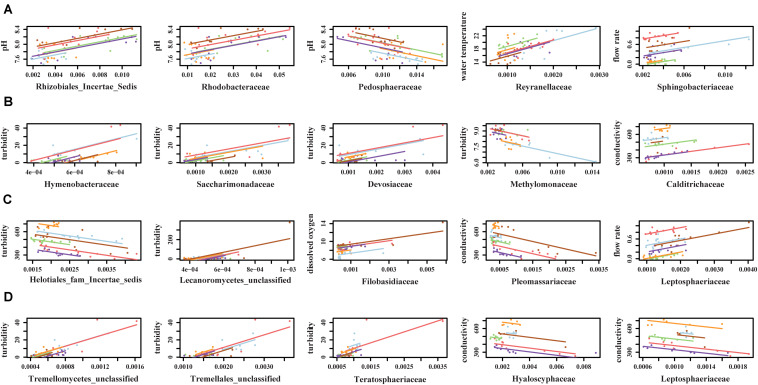
Top five **(A)** bacterial families from sediment fractions, **(B)** bacterial families from water fractions, **(C)** fungal families from sediment fractions, and **(D)** fungal families from water fractions that exhibited strongest correlation with environmental factors. Only families that were detected in relative abundance of 2.5% or higher were included in the repeated measures correlation analysis.

Canonical correspondence analysis results showed that different environmental factors were associated with the composition of microbial communities (Figure 6). Conductivity (*p* = 0.001), pH (*p* = 0.001), and flow rate (*p* = 0.001) were associated with bacterial community composition in sediment fractions (Figure 6), however, they were not associated with bacterial or fungal community composition in water fractions. Turbidity (*p* = 0.023) and conductivity (*p* = 0.004) were associated with fungal communities’ composition in water fractions (Figure 6). Including the sampling site as a within-group factor to account for repeated measures study design (i.e., sampling site and month of sampling) indicated that the sampling site has an effect on the discovered association between environmental factors on microbial communities’ composition. Thus, the relationship between environmental factors and the abundance of different families was tested using repeated measures correlation method. The five families found in a relative abundance of 2.5% or higher in the sediment fractions and exhibited the strongest correlation with individual environmental factors are shown in Figure 7. These included an unassigned families from Rhizobiales and *Rhodobacteraceae* that were positively correlated with the sample pH, and *Pedosphaeraceae* that was negatively correlated with the sample pH. In water fractions, *Reyranellaceae* was positively correlated with water temperature, *Sphingobacteriaceae* was positively correlated with the flow rate, and *Hymenobacteraceae*, *Saccharimonadaceae*, *Devosiaceae* were positively correlated with the sample turbidity. Among fungal families in sediment fractions, an unassigned family under Helotiales and *Pleomassariaceae* were negatively correlated with turbidity, and unclassified families under Lecanoromycetes, *Filobasidiaceae*, and *Leptosphaeriaceae* were positively correlated with turbidity, dissolved oxygen, and flow rate, respectively. An unclassified family under Tremellomycetes, another unclassified family under Tremellales, and *Teratosphaeriaceae* found in water fractions were positively correlated with the turbidity. In contrast, *Hyaloscyphaceae* and *Leptosphaeriaceae* found in water fractions were negatively correlated with the conductivity of the water samples. Change in physiochemical water quality over the course of the study and correlation between physicochemical water quality parameters are reported in Supplementary Figures S4, S6 of Weller et al. (2020), respectively, while associations between pathogen presence and the physiochemical factors are identified and ranked in Supplementary Figure S8 of Weller et al. (2020). Likelihood of both *Salmonella* and *L. monocytogenes* were both associated with turbidity, while *L. monocytogenes* was associated with conductivity, dissolved oxygen, pH, and water temperature.

### Association Between the Presence of Isolated *Salmonella* and *Listeria* Monocytogenes and Specific Microbial or Fungal Families

**FIGURE 8:**
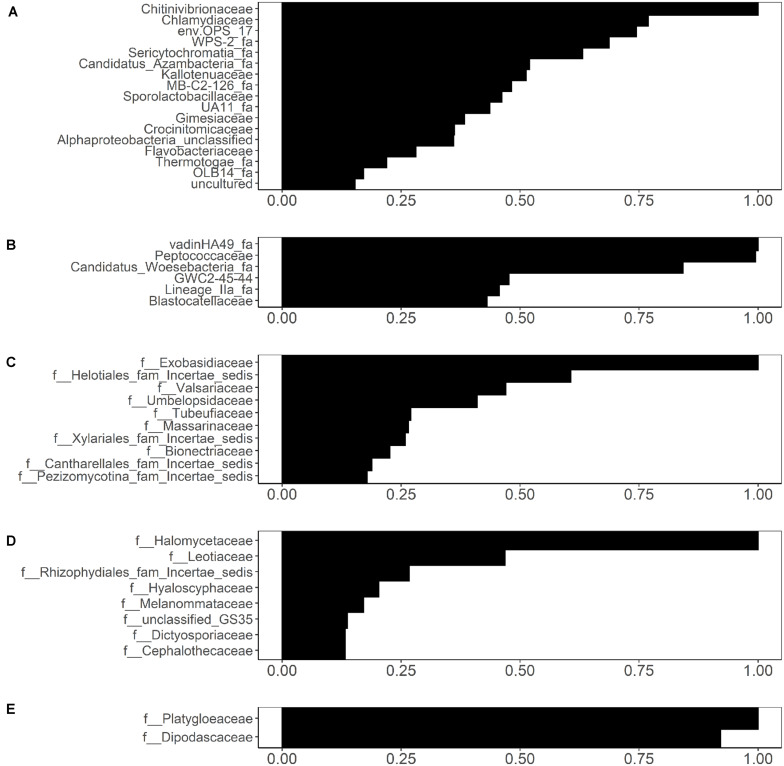
Bacterial families identified through conditional variable importance as associated with **(A)** the presence of isolated *Salmonella* in sediment fractions, **(B)** the presence of isolated *Salmonella* in water fractions, and fungal families identified as associated with **(C)** the presence of isolated *Salmonella* in sediment fractions, **(D)** presence of isolated *Salmonella* in water fractions, and **(E)** presence of isolated *Listeria monocytogenes* in sediment fractions.

While the UniFrac-based PCoA clustering (Supplementary Figure S1) and PERMANOVA (Supplementary Table S9) did not indicate association between microbial community composition and *Salmonella* and *L. monocytogenes* isolation, random forest analysis identified several families that were weakly associated with *Salmonella* and *L. monocytogenes* isolation (Figure 8). Associations were identified between 17 bacterial families in sediment fractions and *Salmonella* isolation (Figure 8A). Of these, *Chitinivibrionaceae*, *Chlamydiaceae*, env.OPS_17 and WPS-2 (candidate taxa) were the four most informative families based on the variable importance measures. In water fractions, associations were identified between 6 bacterial families (VadinHA49, *Peptococcaceae*, Candidatus_Woesebacteria_fa, GWC2-45-44, Lineage_lla_fa, and *Blastocatellaceae*) and *Salmonella* isolation (Figure 8B). In terms of fungal families, associations were identified between 10 fungal families in sediment fractions and *Salmonella* isolation (Figure 8C). Of these, *Exobasidiaceae*, *Valsariaceae*, unidentified family under Helotiales (Helotiales_fam_Incertae_sedis), and *Umbelopsidaceae* were the four top-ranked families based on the variable importance measures (Figure 8C). Fungal families *Halomycetaceae*, *Leotiaceae*, and an unidentified family in Rhizophydiales (Rhizophydiales_fam_Incertae_sedis) were associated with *Salmonella* isolation in water fractions (Figure 8D). Only fungal families *Platygloeaceae* and *Dipodascaceae* identified in sediment fractions were associated with the presence of *L. monocytogenes* in water samples (Figure 8E). Sampling month and site were included as variables in all analyses. In all five analyses, variable importance of sampling month and site were identified as either zero or negative, indicating that the sampling site and month did not have an effect on the random forest-based discovery of microbial families associated with the presence/absence of *Salmonella* and/or *L. monocytogenes.* However, this does not indicate the lack of association between pathogen isolation and the time in the year, since one of the top-ranked factors associated with the isolation of *Salmonella* and *L. monocytogenes* reported in a previous study was a day in a year (Weller et al., 2020). Indeed, when viewed in the context of Weller et al. (2020) finding, this result is indicative of the effect that time unit (month, day) and factor type [continuous (e.g., day of the year), or categorical (e.g., month)] can have on results and data interpretation.

## Discussion

Recent foodborne outbreaks associated with fresh produce that were traced back to the potential use of contaminated water for produce production demonstrate the need for improved strategies for monitoring food safety hazards in agricultural waters (Gelting et al., 2011; Mody et al., 2011; FDA, 2019a). As previously discussed, the relationship between indicators of fecal contamination such as *E. coli* and foodborne pathogen presence in agricultural water appears to vary within and between surface water sources (Haley et al., 2009; Draper et al., 2016; Henry et al., 2016). Thus, alternative strategies for identifying when and where food safety hazards are likely to be present in surface water sources used for produce production are needed. Microbial community composition characterization may be able to elucidate potential alternatives, such as taxa whose presence are associated with a specific pathogen and that can be targeted for testing, or patterns of taxa presence/absence. In order to develop metagenomics-based approaches, there is a need for better understanding of the variability in the surface water microbial communities, and of potential relationships between the surface water microbial communities, environmental conditions, and the presence of key microbial food safety hazards. The study presented here provides data that can help address these knowledge gaps, particularly in Northeast United States. However, it is important to note that this is a small study characterizing just six streams and 68 samples, and as such functions as a proof-of-concept study to generate hypotheses regarding potential associations and trends. Future studies that sample more streams, over a larger time period and in multiple growing regions are need to determine if the associations and trends identified here are reproducible and useful for the development of alternatives to *E. coli*-based water quality tests.

### Microbial Communities Differed Between Sediment and Water Fractions, Suggesting That the Amount of Soil Particles Present in a Water Sample May Affect the Microbial Composition of a Water Sample

Previous studies that investigated microbial communities in water mostly used grab samples that provide a snapshot of microbial water quality at the moment of sample collection (Staley et al., 2013; Van Rossum et al., 2015; Ghaju Shrestha et al., 2017; Hassell et al., 2018; Cagle et al., 2019; Teachey et al., 2019). Here, we used Moore swabs, which act as a filter and capture microorganisms that flow through a stream during the 24 h the swab is deployed. Thus, unlike past studies which filtered the grab samples through modified Moore swab, we separated sediment particles by centrifugation and filtered the remaining water fraction to capture microorganisms that were not attached to soil particles and did not readily sediment during centrifugation due to their larger size. The advantage of the used Moore swab is that it can be easily utilized since it does not require collection of a large volume of water nor advanced equipment for filtration which is required when using modified Moore swabs (Bisha et al., 2011; Weller et al., 2019).

Similar levels of estimated and observed richness in water and sediment fractions found in our study suggest that sample fractionation by centrifugation did not reduce microbial richness in water fractions. However, the taxa that remained in the water fraction were significantly different compared to taxa that were collected in sediment fraction. Previous studies of microbial communities in the Mississippi River and Baltic Sea also found distinct microbial communities in sediments and water, although their approach to sample fractionation was different (Jackson et al., 2014; Rieck et al., 2015; Payne et al., 2017). Similar to our study, these studies reported Actinobacteria, Proteobacteria, and Bacteroidetes as highly abundant in both water and sediment fractions (Staley et al., 2013; Van Rossum et al., 2015; Payne et al., 2017; Wang et al., 2019). Overall, differences in microbial composition between sediment and water fraction in this and other studies makes logical sense since past studies have shown that different microbial taxa have differing abilities to attach to suspended sediment in water (Muirhead et al., 2006; Hemkemeyer et al., 2018). For instance, in a run-off simulation conducted by Muirhead et al. (2006), *E. coli* predominantly attached to particles smaller than 2 μm. Furthermore, Fries et al. reported that an average of 38% of *E. coli* and *Enterococcus* spp. detected in Neuse River Estuary in eastern North Carolina were associated with particles capable of settling out of the water column (Stephen et al., 2006). The sediment compartments, including the bed and suspended sediment have been shown to contain higher loads of microorganisms compared to the water, suggesting that a stream bed may serve as a reservoir for persistence of pathogens (Droppo et al., 2009). Taken together, the amount and type of sediment particles suspended in a water column may affect its microbial composition, and potentially also the ability to detect specific target microorganisms, such as foodborne pathogens. Thus, heavy rain or other conditions that can increase the amount of suspended sediment in the water likely will have an effect on the water microbial community composition as well as the observed richness in water fraction. Identification of significant associations between the flow rate (a proxy for rainfall upstream of the sample collection site) and microbial community composition, and between turbidity and microbial community composition in the study reported here support this hypothesis.

We found *Enterobacteriaceae*, including the genera *Yersinia* and *Thorsellia* in significantly higher relative abundance in water fractions compared to sediment fractions. *Enterobacteriaceae* are commonly used as an indicator of fecal contamination, as this family contains multiple enteric foodborne pathogens, including *Yersinia enterocolitica* (Mossel and Struijk, 1995; Duguma et al., 2013) and increase in their relative abundance may suggest increased fecal contamination, although microbiome data do not provide for absolute quantification of viable microorganisms belonging to this family. Our findings do suggest, however, that *Enterobacteriaceae* may be more likely detected in a water fraction that remains after removing the sediment solids from a water samples using centrifugation. All water fractions contained detectable levels of *Yersinia* spp. DNA. Representatives of this genus have been isolated from different water sources, including untreated surface water and drinking water (Cheyne et al., 2009a, b). Some *Yersinia* species (i.e., *Y. enterocolitica* and *Y. pseudotuberculosis*) have been detected in feces of wild and domestic animals and are known to cause foodborne illness. For example, Bhaduri (Bhaduri et al., 2005) detected *Y. enterocolitica* in 13% (366/2793) of pig fecal samples collected in the United States and tested in their study. Due to the use of short sequence taxonomic markers, we were not able to carry out confident species-level identification in the present study to evaluate the presence of foodborne pathogen’s DNA. In addition to *Yersinia*, all tested water fractions contained detectable levels of *Thorsellia* spp. This genus has previously been used as an indicator of Culex mosquito larvae presence, since it is one of the constituents of the Culex mosquito gut and larvae microbiome (Duguma et al., 2013, 2017). Kämpfer et al. (2015) also reported isolation of *Thorsellia* spp. from larvae of another mosquito, *Anopheles arabiensis*. These findings suggest a consistent presence of mosquitoes in sampled water streams during summer months.

Samples collected from different streams shared a core microbial community, although their composition in terms of microbial relative abundances differed significantly among streams. We observed distinct microbial community composition in samples collected from different streams over 3-month period. This suggests that streams or sampling sites at streams have signature core microbial communities. Deviations from individual stream’s signature microbial communities may therefore be further explored as an indicator of increased risk for introduction of microbial food safety hazards (e.g., fecal contaminants) due to changes in weather, agricultural activity in the watershed or other factors. The watershed land use has previously been associated with the water microbiome composition. Specifically, the agriculturally affected sites that were nutrient-rich were shown to have distinct microbial communities compared to sites affected by urban areas or sites that were unaffected (Van Rossum et al., 2015). In the present study, we collected land use data and compared it qualitatively with microbial community composition. However, further studies are needed to characterize samples collected from a larger number of streams to quantitatively investigate the associations between the watershed land use, composition of microbial communities in water, and microbial quality and safety of water.

### Microbial Families Associated With the Presence of Foodborne Pathogens

A recently published study by Allard et al. had investigated microbial quality and safety of irrigation water used for produce irrigation and characterized water microbiomes in conjunction with microbiological analyses (Allard et al., 2019). However, it had not investigated associations between microbiomes and presence of foodborne pathogens (Allard et al., 2019). Here, we used random forest analysis to identify microbial families associated with *Salmonella* and *L. monocytogenes* isolation. We observed fairly stable core microbiome in each stream over multiple weeks and months, suggesting that sampling on consecutive dates may not have a major effect on random forest analyses. Furthermore, the prevalence of *Salmonella* was 60 ± 7.6% (41/68) (Supplementary Table S3), suggesting that an imbalance between pathogen positive and negative samples was minimum and would hence unlikely cause bias in analyses. Additionally, sampling date and stream were included during random forest analyses and identified that those factors were not associated with the outcome of the model, based on the variable importance being zero or negative. While we identified weak associations between pathogen isolation and multiple microbial families, a number of these families have not been well characterized to date. Nevertheless, some of the identified families, such as *Chlamydiaceae* are represented by known pathogens of mammals, birds, cats, guinea pigs (Brown et al., 2016). Due to a small sample size used in the study reported here, further research focused on investigation of associations and/or predictive power of microbial families identified here is needed to explore their potential for use as microbial indicators of inadequate microbial quality and safety of agricultural waters.

## Conclusion

This study provides baseline data characterizing bacterial and fungal microbial communities in New York agricultural water. Using our approach to sample fractionation into sediment and water fractions, we showed significant differences in microbial community compositions among different sample fractions, indicating that the amount of sediment in a collected water sample likely affects the discovered microbial composition. Furthermore, we identified significant associations between the flow rate (a proxy for rainfall upstream) and microbial community composition, and between turbidity and microbial community composition. Future studies should therefore carefully consider sampling approaches, including sample collection and processing methods, as well as weather prior to sample collection, to improve the sensitivity for detection of target microorganisms. Our study also suggests the existence of microbial community composition fingerprints specific to individual streams or sampling sites at streams. This warrants further investigation into whether perturbances in these microbial community fingerprints may be indicative of increased microbial risk. The machine learning data analyses applied in this study identified specific families weakly associated with the presence of *Salmonella* and *L. monocytogenes*, however, these findings need to be validated in future studies investigating the strength of their association with the presence of foodborne pathogens in samples collected from a greater number of streams.

## Data Availability Statement

The dataset generated for this study is available in the NCBI Sequence Read Archive database under the BioProject accession number PRJNA528961 (https://www.ncbi.nlm.nih.gov/bioproject/PRJNA528961/). Scripts used for bioinformatics and statistical analyses are available in GitHub repository (https://github.com/tuc289/SurfaceWaterMicrobiome).

## Author Contributions

TC extracted DNA, carried out the PCR amplification, analyzed the sequencing data, carried out the statistical analysis, and drafted and revised the manuscript. DW collected and processed the water samples, conducted physicochemical measurements, acquired metadata, and contributed to the data analysis and manuscript revision. JK conceived the study, acquired funding, and revised the manuscript. All authors contributed to the article and approved the submitted version.

## Conflict of Interest

The authors declare that the research was conducted in the absence of any commercial or financial relationships that could be construed as a potential conflict of interest.
