# Supplementary material for: The Composition of Microbial Communities in Six Streams, and Its Association With Environmental Conditions, and Foodborne Pathogen Isolation
Source: Front Microbiol. 2020 Jul 29;11:1757. doi: 10.3389/fmicb.2020.01757 (PMC7403445; doi:10.3389/fmicb.2020.01757)
Supplement: Supplementary file 1 [file Data_Sheet_1.docx]

Supplementary Material

**The composition of microbial communities in six streams, and its association with environmental conditions, and foodborne pathogen isolation**

Taejung Chung^1,2^, Daniel L. Weller^3,4^, Jasna Kovac^1,2^

^1^ Department of Food Science, The Pennsylvania State University, University Park, Pennsylvania, PA 16802, USA

^2^ Microbiome Center, Huck Institutes of the Life Sciences, The Pennsylvania State University, University Park, PA 16802, USA

^3^ Department of Food Science, Cornell University, Ithaca, NY 14853, USA

^4^Current Address: Department of Biostatistics and Computational Biology, University of Rochester, Rochester, NY 14642, USA

**Supplementary Figures**

**Figure S1.** Principal coordinate analysis (PCoA) based on the UniFrac distances. Samples are color-coded based on the presence or absence of (A) *Salmonella* spp. and (B) *Listeria* *monocytogenes* detected using enrichment methods. Blue color indicates the presence of a pathogen and red color indicates the absence of a pathogen. The dots denote a sediment fraction of a sample and triangles denote a water fraction of a sample. Black dots indicate samples for which microbiological detection has not been carried out (i.e., not tested).

**
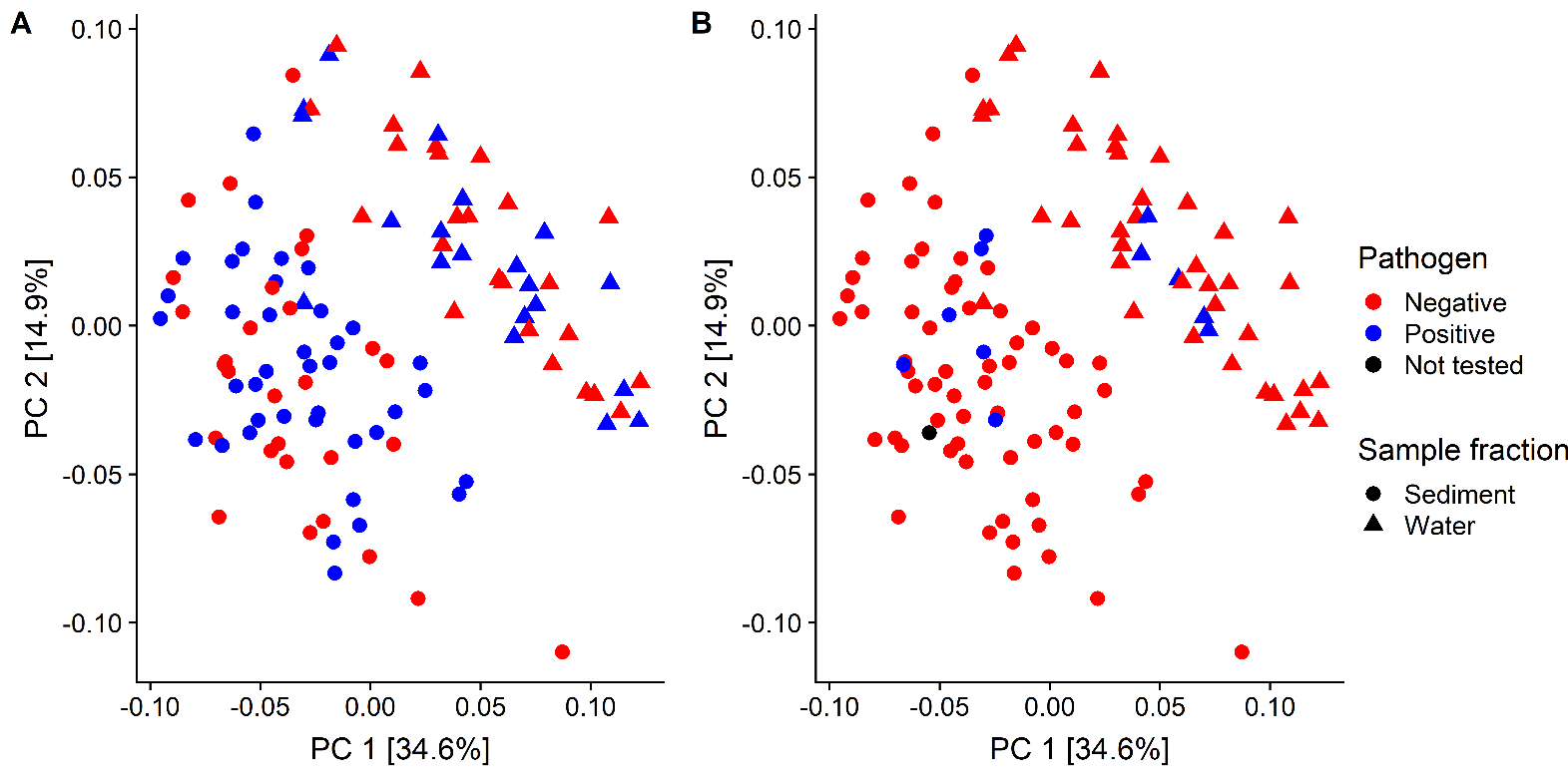
**

**Supplementary Tables**

**Table S1.** Water quality, weather, and hydrological parameters considered in the study reported here. Data were generated by averaging measurements taken when the Moore swab was placed in the waterway and 24 h later when the Moore swab was collected.

| Factor | Minimum | Maximum | Mean | Median | 1st Quartile | 2nd Quartile |
| --- | --- | --- | --- | --- | --- | --- |
| Average Air Temperature (°C) | 14.3 | 32.7 | 22.2 | 21.6 | 19.8 | 24.3 |
| Average Conductivity (uS/cm) | 252 | 734 | 485 | 482 | 364 | 566 |
| Average Dissolved Oxygen (mg/L) | 6.12 | 14.27 | 8.41 | 8.42 | 7.74 | 9.06 |
| Average Flow Rate (m/s) | 0 | 1.1 | 0.4 | 0.3 | 0.1 | 0.6 |
| Average pH | 7.47 | 8.44 | 7.91 | 7.88 | 7.64 | 8.17 |
| Average Turbidity (NTU) | 1.3 | 378.3 | 13.2 | 5.9 | 2.7 | 10.6 |
| Average Water Temperature (°C) | 13.4 | 24.4 | 18.1 | 18.2 | 16.2 | 19.7 |

**Table S2.** SRR accession numbers for sequences of collected samples.

| Sample | Site | SRR accession (16S rDNA, sediment) | SRR accession (16S rDNA, water)^a^ | SRR accession (ITS, sediment) | SRR accession (ITS, water) |
| --- | --- | --- | --- | --- | --- |
| A1 | A | SRR9941240 | N/A | SRR9941241 | N/A |
| A3 | A | SRR9941242 | SRR9941264 | SRR9941251 | SRR9941085 |
| A5 | A | SRR9941262 | SRR9941158 | SRR9941263 | SRR9941219 |
| A7 | A | SRR9941106 | N/A | SRR9941107 | N/A |
| A27 | A | SRR9941108 | N/A | SRR9941109 | N/A |
| A29 | A | SRR9941102 | SRR9941104 | SRR9941103 | SRR9941105 |
| A31 | A | SRR9941100 | N/A | SRR9941101 | N/A |
| A33 | A | SRR9941127 | SRR9941129 | SRR9941126 | SRR9941128 |
| A43 | A | SRR9941131 | SRR9941133 | SRR9941130 | SRR9941132 |
| A45 | A | SRR9941125 | N/A | SRR9941124 | N/A |
| A47 | A | SRR9941161 | SRR9941159 | SRR9941162 | SRR9941160 |
| A49 | A | SRR9941165 | SRR9941163 | SRR9941166 | SRR9941164 |
| B15 | B | SRR9941156 | N/A | SRR9941157 | N/A |
| B17 | B | SRR9941143 | N/A | SRR9941142 | N/A |
| B19 | B | SRR9941141 | SRR9941139 | SRR9941140 | SRR9941138 |
| B21 | B | SRR9941137 | N/A | SRR9941136 | N/A |
| B25 | B | SRR9941135 | SRR9941169 | SRR9941134 | SRR9941170 |
| B34 | B | SRR9941171 | SRR9941173 | SRR9941172 | SRR9941174 |
| B36 | B | SRR9941175 | SRR9941167 | SRR9941176 | SRR9941168 |
| B37 | B | SRR9941194 | SRR9941196 | SRR9941193 | SRR9941195 |
| B40 | B | SRR9941190 | SRR9941192 | SRR9941189 | SRR9941191 |
| B42 | B | SRR9941198 | SRR9941227 | SRR9941197 | SRR9941228 |
| B44 | B | SRR9941225 | SRR9941223 | SRR9941226 | SRR9941224 |
| B46 | B | SRR9941221 | SRR9941113 | SRR9941222 | SRR9941231 |
| C8 | C | SRR9941075 | SRR9941073 | SRR9941074 | SRR9941072 |
| C10 | C | SRR9941110 | SRR9941077 | SRR9941087 | SRR9941076 |
| C12 | C | SRR9941220 | SRR9941086 | SRR9941144 | SRR9941155 |
| C14 | C | SRR9941096 | N/A | SRR9941099 | N/A |
| C25 | C | SRR9941081 | N/A | SRR9941082 | N/A |
| C27 | C | SRR9941083 | SRR9941261 | SRR9941084 | SRR9941112 |
| C29 | C | SRR9941285 | N/A | SRR9941284 | N/A |
| C31 | C | SRR9941283 | SRR9941289 | SRR9941282 | SRR9941288 |
| C35 | C | SRR9941287 | SRR9941281 | SRR9941286 | SRR9941280 |
| C44 | C | SRR9941256 | SRR9941095 | SRR9941257 | SRR9941259 |
| C45 | C | SRR9941252 | SRR9941258 | SRR9941253 | SRR9941250 |
| C47 | C | SRR9941254 | SRR9941249 | SRR9941255 | SRR9941094 |
| D10 | D | SRR9941093 | N/A | SRR9941092 | N/A |
| D12 | D | SRR9941089 | N/A | SRR9941088 | N/A |
| D14 | D | SRR9941091 | SRR9941098 | SRR9941090 | SRR9941097 |
| D27 | D | SRR9941070 | N/A | SRR9941071 | N/A |
| D29 | D | SRR9941068 | N/A | SRR9941069 | N/A |
| D31 | D | SRR9941066 | N/A | SRR9941067 | N/A |
| D33 | D | N/A | SRR9941064 | N/A | SRR9941065 |
| D36 | D | SRR9941078 | SRR9941154 | SRR9941079 | SRR9941153 |
| D38 | D | SRR9941152 | SRR9941150 | SRR9941151 | SRR9941149 |
| D40 | D | SRR9941148 | SRR9941146 | SRR9941147 | SRR9941145 |
| D42 | D | SRR9941114 | SRR9941116 | SRR9941115 | SRR9941117 |
| E1 | E | SRR9941118 | SRR9941120 | SRR9941119 | SRR9941121 |
| E4 | E | SRR9941122 | SRR9941202 | SRR9941123 | SRR9941201 |
| E6 | E | SRR9941204 | SRR9941206 | SRR9941203 | SRR9941205 |
| E9 | E | SRR9941208 | N/A | SRR9941207 | N/A |
| E11 | E | SRR9941200 | SRR9941181 | SRR9941199 | SRR9941182 |
| E13 | E | SRR9941179 | SRR9941185 | SRR9941180 | SRR9941186 |
| E15 | E | SRR9941183 | SRR9941177 | SRR9941184 | N/A |
| E20 | E | SRR9941178 | SRR9941267 | SRR9941268 | SRR9941266 |
| E22 | E | SRR9941265 | SRR9941271 | SRR9941272 | SRR9941270 |
| E24 | E | SRR9941269 | SRR9941275 | SRR9941276 | SRR9941236 |
| E26 | E | SRR9941237 | SRR9941239 | SRR9941238 | SRR9941232 |
| F8 | F | SRR9941233 | N/A | SRR9941234 | N/A |
| F10 | F | SRR9941235 | N/A | SRR9941244 | N/A |
| F12 | F | SRR9941245 | SRR9941210 | SRR9941209 | SRR9941211 |
| F14 | F | SRR9941212 | N/A | SRR9941213 | N/A |
| F25 | F | SRR9941214 | N/A | SRR9941215 | N/A |
| F27 | F | SRR9941216 | N/A | SRR9941217 | N/A |
| F31 | F | SRR9941218 | N/A | SRR9941247 | N/A |
| F34 | F | SRR9941246 | SRR9941111 | SRR9941260 | SRR9941243 |
| F36 | F | SRR9941248 | SRR9941063 | SRR9941080 | SRR9941230 |
| F38 | F | SRR9941229 | SRR9941188 | SRR9941187 | SRR9941273 |
| F40 | F | SRR9941274 | SRR9941279 | SRR9941278 | SRR9941277 |

^a^ N/A, not applicable; sample was not sequenced due to insufficient quantity of DNA.

**Table S3.** Pathogen prevalence in Moore swab samples collected from different sampling sites.

| Site | *Salmonella* spp. | *L. monocytogenes* |
| --- | --- | --- |
| A | 66 % (8/12) | 8 % (1/12) |
| B | 50 % (6/12) | 0 % (0/12) |
| C | 58 % (7/12) | 27 % (3/11) |
| D | 70 % (7/10) | 0 % (0/10) |
| E | 54 % (6/11) | 18 % (2/11) |
| F | 63 % (7/11) | 0 % (0/11) |
| Total | 60 % (41/68) | 9% (6/67) |

**Table S4.** Differences in bacterial and fungal communities between sediment and water fractions of water samples.

| Communities^a^ | Df^b^ | SS^c^ | MS^d^ | F.Model^e^ | p-value |
| --- | --- | --- | --- | --- | --- |
| Bacterial | | | | | |
| Sample fraction | 1 | 0.25108 | 0.25108 | 30.538 | 0.0001 |
| Residuals | 111 | 0.91264 | 0.00822 |  |  |
| Total | 112 | 1.16373 |  |  |  |
| Fungal | | | | | |
| Sample fraction | 1 | 0.172 | 0.172 | 16.402 | 0.0001 |
| Residuals | 110 | 1.1536 | 0.0104 |  |  |
| Total | 111 | 1.3256 |  |  |  |

^a^ Sample fraction, sediment or water fraction.

^b^ Degree of freedom.

^c^ Sum of square.

^d^ Mean square.

^e^ Pseudo F statistic.

**Table S5.** Fold change in relative abundance of bacterial families between water and sediment fractions.

| Family | Log fold change  sediment/water | |
| --- | --- | --- |
| Arcobacteraceae | | 2.291493299 |
| Clade_III | | 2.275488021 |
| Sporichthyaceae | | 2.184772851 |
| Alteromonadaceae | | 2.000064018 |
| Mycobacteriaceae | | 1.828176382 |
| NS11-12_marine_group | | 1.801716926 |
| Pseudohongiellaceae | | 1.767151835 |
| uncultured_fa | | 1.675336006 |
| Lentisphaeraceae | | 1.266964915 |
| Reyranellaceae | | 1.208652737 |
| Gallionellaceae | | 1.163764837 |
| Cyanobiaceae | | 1.159714522 |
| Thiovulaceae | | 1.088938 |
| Terrimicrobiaceae | | 1.027003887 |
| Salinivirgaceae | | -1.020464656 |
| SC-I-84 | | -1.021029589 |
| PLTA13_fa | | -1.027882693 |
| Rhodanobacteraceae | | -1.070674655 |
| Nitrospiraceae | | -1.107034442 |
| Clostridiaceae | | -1.136973617 |
| MB-A2-108_fa | | -1.141584156 |
| Run-SP154_fa | | -1.177356718 |
| B1-7BS | | -1.199111698 |
| Bacteroidetes_vadinHA17 | | -1.230688093 |
| P9X2b3D02_fa | | -1.261426089 |
| Ignavibacteriaceae | | -1.265557287 |
| KD4-96_fa | | -1.269854782 |
| Blastocatellaceae | | -1.312373517 |
| FW113_fa | | -1.329177854 |
| Nakamurellaceae | | -1.380642054 |
| Subgroup_7_fa | | -1.430565355 |
| Subgroup_17_fa | | -1.492305634 |
| Gaiellaceae | | -1.500332115 |
| Xanthomonadaceae | | -1.573551176 |
| CCM19a_fa | | -1.584628818 |
| Steroidobacteraceae | | -1.67254245 |
| Dehalococcoidia_fa | | -1.749934075 |
| PHOS-HE36 | | -2.271623251 |
| Halieaceae | | -2.36982405 |
| MB-C2-126_fa | | -2.514422295 |
| AT-s2-59_fa | | -3.041892169 |

**Table S6.** Fold change in relative abundance of fungal families between water and sediment fractions.

| Family | Log fold change sediment/water |
| --- | --- |
| Microstromatales_fam_Incertae_sedis | 2.733666208 |
| unclassified_Arthoniomycetes | 1.716370609 |
| unclassified_GS11 | 1.524818924 |
| Rozellomycotina_fam_Incertae_sedis | 1.457733658 |
| Rozellomycotina_cls_Incertae_sedis_unclassified | 1.269062546 |
| Diatrypaceae | 1.040456083 |
| unclassified_Rozellomycota | 1.040047315 |
| Trichosphaeriaceae | -1.018897507 |
| unclassified_Thelebolales | -1.202714178 |
| Botryosphaeriaceae | -1.24297652 |
| Candelariaceae | -1.283547613 |
| Cladosporiaceae | -1.492751691 |
| Teloschistaceae | -1.699431512 |

**Table S7.** Pairwise comparison of microbial alpha diversity at a family level in samples collected from different streams.

| Bacterial Communities | | | | Fungal Communities | | | | |
| --- | --- | --- | --- | --- | --- | --- | --- | --- |
| Sediment fraction | | Water fraction | | | Sediment fraction | | Water Fraction | |
| Sampling Site | Inverse  Simpson | Sampling site | Inverse  Simpson | | Sampling Site | Inverse  Simpson | Sampling site | Inverse  Simpson |
| A vs B | 1 | A vs B | 0.3239 | | A vs B | 1 | A vs B | 0 |
| A vs C | 1 | A vs C | 1 | | A vs C | 1 | A vs C | 0.2276 |
| A vs D | 0.0185 | A vs D | 1 | | A vs D | 1 | A vs D | 0.1091 |
| A vs E | 1 | A vs E | 0.7936 | | A vs E | 1 | A vs E | 0.0234 |
| A vs F | 1 | A vs F | 1 | | A vs F | 1 | A vs F | 1 |
| B vs C | 0.6782 | B vs C | 1 | | B vs C | 1 | B vs C | 0.2818 |
| B vs D | 0.0076 | B vs D | 0.0003 | | B vs D | 1 | B vs D | 0.837 |
| B vs E | 1 | B vs E | 1 | | B vs E | 1 | B vs E | 1 |
| B vs F | 1 | B vs F | 1 | | B vs F | 1 | B vs F | 0.4626 |
| C vs D | 1 | C vs D | 0.0723 | | C vs D | 1 | C vs D | 1 |
| C vs E | 0.0134 | C vs E | 1 | | C vs E | 1 | C vs E | 1 |
| C vs F | 1 | C vs F | 1 | | C vs F | 1 | C vs F | 1 |
| D vs E | 0 | D vs E | 0.0013 | | D vs E | 1 | D vs E | 1 |
| D vs F | 0.0442 | D vs F | 0.395 | | D vs F | 1 | D vs F | 1 |
| E vs F | 1 | E vs F | 1 | | E vs F | 1 | E vs F | 1 |

**Table S8.** Percent of the upstream watershed area that was classified as developed, natural (i.e., forest, grassland., shrubland or wetland), pasture and cropland, for each watershed and distance class.

^a^ Open water and barren land comprised less than 2% of each watershed 0-250 m upstream of the sampling site.

| Stream | Percent of Upstream Area Classified in Each Land Cover Class | | | | | | | | | | | | | | | | |
| --- | --- | --- | --- | --- | --- | --- | --- | --- | --- | --- | --- | --- | --- | --- | --- | --- | --- |
|  | 0-250 m Upstream of Site^a^ | | | | 0-500 m^b^ | | | | 0-1000 m^c^ | | | | Whole Watershed^d^ | | | | |
|  | Developed | Natural | Pasture | Crop | Developed | Natural | Pasture | Crop | Developed | Natural | Pasture | Crop | Developed | Natural | Pasture | Crop | Total Area (km2) |
| A | 35.6 | 30.0 | 34.4 | 0.0 | 17.3 | 25.8 | 37.2 | 19.7 | 6.2 | 25.0 | 28.1 | 40.3 | 3.1 | 41.4 | 21.8 | 33.7 | 22.3 |
| B | 41.2 | 58.8 | 0.0 | 0.0 | 25.1 | 74.9 | 0.0 | 0.0 | 8.6 | 91.4 | 0.0 | 0.0 | 3.9 | 63.8 | 23.0 | 9.1 | 176.1 |
| C | 6.5 | 0.0 | 56.9 | 36.6 | 3.6 | 20.9 | 40.9 | 34.6 | 8.8 | 24.4 | 38.3 | 25.2 | 4.1 | 42.8 | 31.6 | 21.2 | 143.2 |
| D | 47.8 | 46.3 | 5.7 | 0.0 | 16.1 | 37.7 | 14.3 | 0.0 | 6.2 | 40.3 | 22.7 | 14.7 | 8.1 | 37.0 | 18.3 | 35.3 | 40.5 |
| E | 16.4 | 17.6 | 66.1 | 0.0 | 28.7 | 15.4 | 48.5 | 6.7 | 14.1 | 44.3 | 26.2 | 15.0 | 3.4 | 77.4 | 6.9 | 11.8 | 25.4 |
| F | 9.7 | 3.6 | 79.7 | 6.9 | 11.8 | 5.1 | 60.1 | 22.9 | 6.7 | 15.2 | 33.9 | 44.2 | 2.7 | 71.4 | 16.9 | 7.0 | 19.5 |

^b^ Open water and barren land comprised less than 2% of each watershed 0-250 m upstream of the sampling site, with the exception of Stream D. For Watershed D 0-500 m upstream for the sampling site, 6.6% was open water and 25.4% was barren land.

^c^ Open water and barren land comprised less than 2% of each watershed 0-250 m upstream of the sampling site, with the exception of Stream D and C. For Watershed C 0-1000 m upstream for the sampling site, 3.3 % was open water and <1% was barren land. For Watershed D 0-1000 m upstream for the sampling site, 4.1% was open water and 12.1% was barren land.

^d^ Open water and barren land comprised less than 2% of each watershed 0-250 m upstream of the sampling site.

**Table S9.** Statistical differences in microbial community composition among samples positive for *Salmonella* or *Listeria monocytogenes.*

| Foodborne Pathogen | Bacterial Communities^a^ | | Fungal Communities^a^ | |
| --- | --- | --- | --- | --- |
|  | Sediment Fraction | Water Fraction | Sediment Fraction | Water Fraction |
| *Salmonella* spp. | 0.442 | 0.25 | 0.444 | 0.953 |
| *Listeria monocytogenes* | 0.448 | 0.167 | 0.622 | 0.325 |

^a^ P values were obtained using PERMANOVA.
